# Recent advancements in prebiotic oligomers synthesis via enzymatic hydrolysis of lignocellulosic biomass

**DOI:** 10.1080/21655979.2021.2023801

**Published:** 2022-01-16

**Authors:** Reetu Saini, Anil Kumar Patel, Jitendra Kumar Saini, Chiu-Wen Chen, Sunita Varjani, Reeta Rani Singhania, Cheng Di Dong

**Affiliations:** a Department of Marine Environmental Engineering, National Kaohsiung University of Science and Technology, Kaohsiung City, Taiwan; b Department of Microbiology, Central University of Haryana, Mahendergarh, India; c Gujarat Pollution Control Board, Gandhinagar, India

**Keywords:** Prebiotics, oligosaccharides, lignocellulose, enzymatic hydrolysis, functional foods, tailor-made enzyme cocktail

## Abstract

Interest in functional food, such as non-digestible prebiotic oligosaccharides is increasing day by day and their production is shifting toward sustainable manufacturing. Due to the presence of high carbohydrate content, lignocellulosic biomass (LCB) is the most-potential, cost-effective and sustainable substrate for production of many useful products, including lignocellulose-derived prebiotic oligosaccharides (LDOs). These have the same worthwhile properties as other common oligosaccharides, such as short chain carbohydrates digestible to the gut flora but not to humans mainly due to their resistance to the low pH and high temperature and their demand is constantly increasing mainly due to increased awareness about their potential health benefits. Despite several advantages over the thermo-chemical route of synthesis, comprehensive and updated information on the conversion of lignocellulosic biomass to prebiotic oligomers via controlled enzymatic saccharification is not available in the literature. Thus, the main objective of this review is to highlight recent advancements in enzymatic synthesis of LDOs, current challenges, and future prospects of sustainably producing prebiotic oligomers via enzymatic hydrolysis of LCB substrates. Enzyme reaction engineering practices, custom-made enzyme preparations, controlled enzymatic hydrolysis, and protein engineering approaches have been discussed with regard to their applications in sustainable synthesis of lignocellulose-derived oligosaccharide prebiotics. An overview of scale-up aspects and market potential of LDOs has also been provided.

## Graphical Abstract

**Figure:**
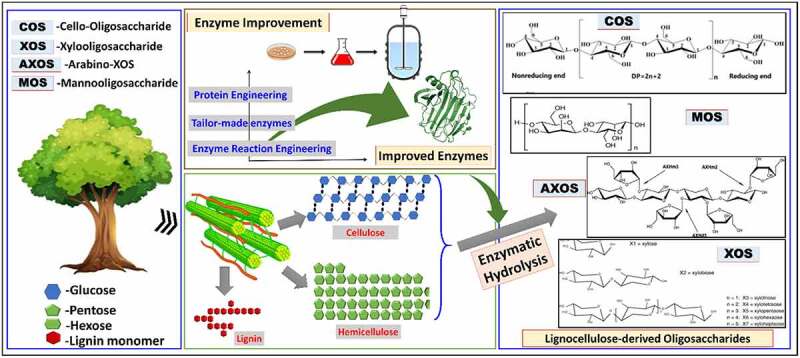

## 1. Introduction

At present, many food and pharma industries are showing increasing interest in developing functional foods having potential health benefits, including reduced risk of diseases. Major target foods for this purpose ‘prebiotics’, also known as ‘nondigestible oligosaccharides’ (NDO)’ or ‘carbohydrates with low degree of polymerization (DP ≤ 10)’ [1]. The credit for the idea of prebiotics goes to Gibson and Roberfroid [2], who stated these as ‘a nondigestible food ingredient that beneficially affects the host by selectively enhance the growth/activity of one or a limited number of bacteria in the colon, and thus improves host health’. However, this definition was later modified to ‘a selectively fermented ingredient that results in specific changes in the composition and/or activity of the gastrointestinal microbiota, thus conferring benefit(s) upon host health’, so as to include other non-carbohydrate compounds as well [3]. Ideally, the prebiotic components should be non-digestible to the host, fermentable by microbes in host’s intestine, and able to selectively enhance growth/activity of the potentially useful microbes [4]. These can be categorized into different types based upon molecular size or DP of constituent carbohydrate [5]. Prebiotics include fructo-oligosaccharides (FOS), galacto-oligosaccharides (GOS), lacto-sucrose, xylo-oligosaccharides (XOS), isomalto-oligosaccharides (IMOS), and soybean oligosaccharides (SOS) [6].

Non-digestible oligosaccharides having prebiotic properties are found in many foods naturally (but in very low amount), including asparagus, sugar beet, garlic, chicory, onion, Jerusalem artichoke, wheat, honey, banana, barley, tomato, rye, soybean, human’s and cow’s milk, peas, beans, etc., and recently, seaweeds and microalgae but in very low amount [7]. However, recent research has shown that prebiotic production is shifting toward the sustainable manufacturing, for which lignocellulosic biomass (LCB) is considered as the most promising source, as this carbohydrate polymer is enormously available on earth. LCB feedstocks majorly include agricultural residues such as straws (wheat, paddy, mustard), stalks (cotton, mustard), and bagasse (sugarcane and sweet sorghum), forestry wastes, municipal solid wastes and industrial wastes as well [8]. Each year, such agro-residues are produced in surplus and have to be burnt to get rid of them. A sustainable alternative for their management and resource recovery could be conversion of these waste biomass resources into valuable products such as prebiotic oligosaccharides. Due to the presence of high carbohydrate content, lignocellulosic biomass (LCB) has shown greatest potential in cost-effective and sustainable production of many useful products, such as biofuels, biochemicals and other value-added products, including prebiotics. Three major components of LCB include cellulose, hemicellulose and lignin that have a strong cross link with each other, making the plant biomass highly recalcitrant. The glucan (cellulose) and xylan (hemicellulose) components of LCB feedstock can be converted into cello- and xylo-oligosaccharides, respectively. Various LDOs produced during the hydrolysis of heterogeneous hemicellulose component of lignocellulosic biomass include arabino-XOS (AXOS), mannano-oligosaccharide (MOS), arabino-oligosaccharide (AOS), and glucuronosylated-XOS (GXOS) [6]. Lignocellulosic derived oligosaccharides (LDOs) have the same worthwhile properties as other common oligosaccharides, such as short chain carbohydrates digestible to the gut flora but not to humans mainly due to their resistance to the low pH and high temperature [9].

Production of prebiotic oligomer synthesis from LCB can be carried out by enzymatic or thermo-chemical conversion processes, either individually or in combination. Thermo-chemical processes for production of oligosaccharides include the use of heat alone or in conjunction with mild chemicals. In contrast, enzyme-based oligomer production from LCB offers advantages, such as, increased yield due to less production of undesired products, mild operational conditions, and decreased cost, especially when the enzyme-recycling is done [10]. Additionally, sequential use of chemical and enzymatic saccharification of LCB is also an effective method for production of prebiotic oligosaccharides [10–14].

Hydrolytic enzymes involved in LDO production include various hydrolytic and non-hydrolytic or de-branching enzymes including cellulases and various hemicellulases along with some accessory enzymes. Various methods have been reported for enzyme-based oligosaccharide production [1,15,16]. However, each strategy has its own advantages and limitations, and there is no universal method which can be employed for LDO production from various LCBs [1].

Overall, it can be concluded that new or underdeveloped prebiotics are in more demand due to increased awareness about their potential health benefits. Although, enzyme-based biochemical route for LDO production offers several advantages over the thermo-chemical route, comprehensive, and updated information on the conversion of lignocellulosic biomass to prebiotic oligomers via enzymatic saccharification is not available in the literature. Therefore, the main aim of this review is providing a basic understanding of the concepts and recent advancements of enzymatic synthesis of LDOs, and comprehensive assessment of current practices, challenges and prospects of sustainably producing prebiotic oligomers via enzymatic hydrolysis of LCB substrates. Enzyme reaction engineering practices, custom-made enzyme preparations, controlled enzymatic hydrolysis, and protein engineering approaches have also been discussed with regard to their applications in sustainable synthesis of lignocellulose-derived oligosaccharide prebiotics.

## 2. Market potential of prebiotics

The applications of prebiotics are not only limited to humans and these are also used in animal feeds to improve overall health of animals, especially for increased digestion by reducing acidosis to improve milk and meat production. Because of the global demand of such ingredients and awareness of health benefits, it is necessary to manufacture such compounds on large scale by various industries all over the world. Thus, functional foods industrial sector is likely to grow up. The food and beverage (F & B) industries, pharma & healthcare industries, biotech industries, animal-feed industries, etc. are the major sectors involved in prebiotic synthesis [17]. Major global prebiotic manufacturers include: Beneo GmbH, Roquette America Inc., Cargill, Inc., Friesl and Campina Domo, Yakult Industries, Clasado Ltd., Jarrow Formulas, Inc. Abbott Laboratories, Dupont, Friesland Campina, SOLACTIS Group Ltd., and Jarrow Formulas [https://www.industryarc.com/Report/7481/prebiotics-ingredients-market.html; assessed in October 2021].

As per the recent analysis, the worldwide functional prebiotic ingredients market is expected to reach over 9.4 billion USD by 2026, considering a 5-year (2021–2026) annual growth of 8.7%, in comparison to the estimated market potential of 4.5 billion USD during 2020. Among the established prebiotics, the market potential of FOS, inulin and XOS during 2019–20 was worth USD 2.37 billion, 1.4 billion and 99 million, with per annum growth rate of 10, 6.4 and 4.4%, respectively. XOS market is expected to grow close to 130 million USD at 4.4% CAGR by 2025 [https://www.wboc.com/story/44634719/xylooligosaccharides-xos-market-size-in-2021-with-a-cagr-of-44-business-demand-market-share-trend-business-news-business-growth-prime-key-players; assessed in October 2021; https://www.verifiedmarketresearch.com/product/global-fructooligosaccharide-fos-market-size-and-forecast-to-2025/; assessed in October 2021]. This sharp increase is expected mainly due to more awareness of sugar-, fat- and calorie-free healthy diets, health benefits of functional, and fiber-rich foods, specifically in immune stimulation mainly prebiotics, along with concerns of weight management, digestive problems, increasing charm for physical fitness, especially during the COVID19 pandemic period [www.gminsights.com/pressrelease/prebiotics-market-size.; assessed in October 2021].

## 3. Classification and characteristics of prebiotic oligosaccharides

Many microorganisms living inside the human gut (50% noncultivable microbes) play an important role in improving the digestion and overall health of their human hosts, specifically due to their ability to break even those complex food ingredients (carbohydrates and others) that remain undigested in the upper digestive system [6,18,19]. Such microorganisms derive their energy through fermentation of the nondigestible compounds present in the diet, converting them to useful metabolites in the due course [2,18], thereby, improving the overall gut health. There are two ways to foster the health-promoting activities of these microorganisms, either by their intake in live form as a food supplement (probiotic food, pharmaceutical, and health-care products) or by promoting the growth of such already existing microbes through increased dietary intake of non-digestible food ingredients called ‘prebiotics’ [2,6]. Thus, prebiotic food components have the capacity to alter the growth and activity of intestinal microbes, and overall gut microbial community. Additionally, prebiotics can selectively influence gut microbiota, which in turn affect the metabolic functions and absorption of the intestine, suppress pathogenic microbes, modulate human immune system, and make adhesion sites less available to harmful organisms [20,21]. A prebiotic compound should resist the acidic conditions and action of the hydrolytic enzymes in the upper digestive tract and should remain unabsorbed there; should be fermented by the intestinal microbes, acting as a source of energy, and; provide selective stimulation of the growth and activities of the microbes present in intestine, thereby, providing health benefits to hosts [3].

Prebiotics are of many types, majorly belonging to carbohydrate group and a few, also reported as non-carbohydrate prebiotics [22]. The carbohydrate type of prebiotics includes the ‘non-digestible oligosaccharides (NDOs)’, which do not get digested in upper digestive tract due to their non-digestible nature. Therefore, after reaching the colon in intact form, NDOs are fermented by the action of intestinal microorganisms and are converted into volatile fatty acids such as, acetic acid, butyric acid, propionic acid, etc. Amount and type of the fermentation products depend upon the type of gut microbial communities [23]. These fatty acids in turn provide several health benefits such as helping the bowel movement, reducing the level of sugars in blood, etc [2,19,24–27]. Previously, many researchers have shown the beneficial effects of prebiotics on human health in improving the mineral uptake, lipid breakdown/ absorption, and body’s immunity, development of epithelium of intestine, prevention of the onset of cancer of intestine, protection against heart and metabolism related disorders and providing ‘barrier-effect’ against intestinal pathogenic microbiota [22,24,25,28]. Prebiotics not only influence the colon, where these get metabolized through microbial fermentation, but are also able to exert their beneficial effects over other organ systems [29].

Oligosaccharides (OS) have a degree of polymerization (DP) values that are intermediate to that of monomers and polysaccharides (i.e. DP~2 to 10), and may be present in free or the bound forms [30,31]. The anomeric carbons of the monomeric sugars of NDOs provide the characteristics of nonsusceptibility toward intestinal hydrolases and low pH [5,31]. The prebiotic OS are derived in nature, although in a very low quantity, from various plant-, animal-, and microbe-based food sources, including various vegetables, fruits, food grains, milk, seaweeds, and microalgae [32]. Other, types of prebiotics that have also been recently reported to possess health benefits include the pectin-derived oligosaccharides (POS) [33].

Among various compounds reported to possess prebiotic properties so far, only few have been recognized as the established ones, including FOS, GOS, and lactulose. POS, soybean oligosaccharides, C/GOS, XOS, MOS, etc. are categorized as ‘emerging prebiotics’. Besides, there are other compounds also, that are known to have very high potential prebiotic properties, such as maltodextrin, raffinose, arabinose, AXOS, as well as sugar alcohols (mannitol and sorbitol) [31,34]. Various prebiotic compounds have been discussed below and their important characteristics have been summarized in Table 1.

**Table 1.** Characteristics of various types of prebiotic oligosaccharides

Type	Examples of substrates	Properties	Production method	Applications	References
**COS**	Lignocellulosic biomass (Sugarcane straw)	DP-2-6 glucose molecules with β-1, 4-linkages, Water-soluble dietary fibers	Autohydrolysis, Chemical methods, Direct enzymatic hydrolysis Three-enzyme cascade system reported to produce COS: sucrose phosphorylase, cellobiose phosphorylase and cellodextrin phosphorylase	Pharmaceuticals Feed formulations Food applications Bioethanol production	[4,35,127]
**MOS**	Coffee beans, Soybeans, Alfalfa seeds, Ivory nuts, Sugar beets, Cell walls of some fungi, yeasts and bacteria, Roots and tubers of orchids, Legume seeds, Coconut kernel, Palm kernel	Water Soluble, Stable in Aqueous solution	Galactosidase, Galactohydrolase requires for hydrolysis of mannans to produce mannooligosaccharides	Boost animal’s immunity Promoting health of broiler In animals like pigs and broiler, they are potential feed additives	[36,37]
**XOS**	Bengal gram husk, Wheat bran and straw, Spent wood, barley hulls, Brewery spent grains, Almond shells, Bamboo Corn cob.	Xylose moieties linked by β-(1,4) bonds Polymerization degree ranging from 2 to 10 Also known to act as a plant growth regulator	Chemical methods, Autohydrolysis, Thermal process and Direct enzymatic hydrolysis of a susceptible substrate	Antioxidant Gelling agent in food products Beneficial for diabetes, Treatment of arteriosclerosis Reduces total cholesterol & LDL in patients with type 2 diabetes Anti-colon cancer	[12,95]
**AXOS**	Monocotyledonous biomass {(ryegrass pulp (RG) and wheat straw WS)}	A mix of OS constituted by a linear β-(1→4)-D-xylopyranan backbone DP 2–10 molecules of xylose	Pretreatment of biomass followed by Enzymatic hydrolysis	Bacterial growth stimulating response in colon Facilitates nutritional utilization By animals Improve the GI health of humans	[38,82]
**GOS**	Cow’s milk and human’s milk, lactose Soybean seeds	Poorly hydrolyzed and digested in the intestinal tract of gnotobiotic rats Glucose units linked by α1–6 and α1–2 glycosidic bonds	Enzymatically synthesized Using a glucosyl-transferase	Effects on gut health Anti-colon cancer, anti-inflammatory Bowel disease	[18,31,39]
**FOS**	Garlic, Tomato, Onion, Honey, Rye, Barley, Banana Chicory, Asparagus, etc.	Naturally present in plants and regulate plant growth Have DP value ranging from 2 to 10	Commercially, they are being produced by the action of fructosyltransferase or β-fructofuranosidase From microbial sources	Stimulating growth of gut bacteria Activation of human immune system Enhanced mineral absorption in the GI tract Synthesis of B complex vitamin, Reduction of serum Cholesterol, Prevention carcinogenic tumors, Having low calories	[10,31,40]

### 3.1 Galacto-oligosaccharide (GOS)

GOS are ‘a mixture of those substances produced from lactose, comprising between 2 and 8 saccharide units, with one of these units being a terminal glucose and the remaining saccharide units being galactose and disaccharides comprising 2 units of galactose’ [41] GOS act as ideal prebiotic due to their nondigestibility in the upper digestive system, while remaining fermentable to the gut microbes. Therefore, these are also called ‘nondigestible fibers’ or ‘befidiogenic prebiotics’ (supporting growth of *Bifidobacteria* and other related bacterial species) [42,43]. GOS possess β-1,6-linked galactose chains ending with a reducing β-1,4 linked glucose moiety. Both β- and α-linked GOS are available naturally, but the prebiotic effects of the latter have been reported to be very low. Industrially, lactose is used to form β-GOS by the application of β-galactosidase’ glycosyltransferase activity, whereas, α-GOS having the α-1,6 linkages is naturally derived from soybeans [43,44]. Transgalactosylation reaction produces GOS with DP 3–5, called ‘trans-galacto-oligosaccharides or TOS’, where galactose might be joined through β-1,6, 1,3 or 1,4 links [45]. Prebiotic GOS can also be produced using lactose isomer ‘lactulose’ [3]. Raffinose-oligosaccharides (RFO), are the other types of GOS, based on sucrose extension, however, their prebiotic properties (selective stimulation of gut microbes) need extensive investigations [46,47].

For higher production of GOS by transgalactosylation of lactose, very high concentrations of lactose must be used. Microbial cells (e.g. *Kluyveromyces marxianus, Pseudozyma* tsukubaensis, and *Pichia kluyveri*) or the enzymatic reactions (enzyme β-galactosidase derived from *Kluyveromyces lactis* and *Aspergillus oryzae*) can be used for GOS synthesis from lactose or various agro-industrial waste materials (e.g. whey) can also be employed for sustainable GOS production [48–52].

### 3.2 Fructo-oligosaccharide (FOS)

Inulin and FOS are collectively termed ‘fructans,’ and both are constituted of linear chains of fructose joined with β-2,1-links, with a DP of up to 60 and 10, respectively. FOS generally contain glucose moiety at the end. This linkage in both inulin and FOSs cannot be hydrolyzed by the hydrolytic enzymes, thus, giving them the characteristic feature of prebiotics. It has been proven in past that fructans have the characteristic feature of specifically promoting lactic acid bacteria (LAB), but the DP has a significant effect on bacterial fermentation [53,54]. Depending upon the length of fructose chain, gut microbes other than LAB can also be generally stimulated by such prebiotics [7]. FOS are also termed ‘oligofructan’ or ‘oligofructose’ and find applications as artificial sweetener [55].

Naturally, FOS and inulin are derived from many food sources: asparagus, onion, artichokes, chicory, banana, etc. But, due to very less natural synthesis, industrial production employs two methods: enzymatic and chemical (hydrolysis of inulin to FOS) [56]. During enzymatic production, enzymes produced by fungi or bacteria are used. Fungal enzymes are preferred over the bacterial enzymes, due to their higher titer and extracellular nature. Enzyme fructosyl transferases (FTase) cause transfructosylation of sucrose and are preferred for FOS production [57]. FOS synthesis by transfructosylation reaction require use of higher initial quantities of sucrose (~800 g/L). Inulinase is also used to synthesize FOS by endo-hydrolysis of inulin [58]. Enzymatic production of FOS can be carried out industrially by using whole microbial cells or their extracted enzymes. The enzymes can be used freely in the reaction mixture or after their immobilization in suitable matrix.

### 3.3 Xylo-oligosaccharides (XOS)

The nondigestible oligosaccharides XOS are ‘xylose (pentose sugar) derived short (DP 2–10) oligosaccharides that are linked together with β-1,4-linkages’ and are generated from xylan rich compounds. Some researchers also suggest considering the compounds of xylose DP up to 20 as XOS [59]. Xylan, also known as hemicellulose, is the second most abundant natural polysaccharide on earth after cellulose and is a major component of plant cell wall structure. Thus, a sustainable source for XOS production for industrial application is the lignocellulosic biomass, such as agro-industrial residues (bagasse, straw, etc.). XOS find applications as sweetener in food-additives and can be obtained by physical or chemical hydrolysis of LCB and by enzymatic hydrolysis of plant-biomass [60]. Besides, these prebiotic compounds are also present in shoot of bamboo and various fruits and vegetables, as well as milk and honey. XOS exhibit prebiotic properties, by being resistant to hydrolytic enzymes, and supporting the growth and stimulation of gut bacteria, predominantly *Bifidobacterium* sp [61]. Other major health benefits offered by XOS include control of release of insulin by the pancreas, regulating cholesterol in the blood, regulation of procarcinogenic enzymes of the intestine, increased absorption of minerals in large intestine, apart from their antioxidant and anti-inflammatory characteristics [59,62–65].

XOS synthesis can be carried out by chemical, enzymatic, or autohydrolytic methods [65]. Chemical method involves hydrolysis of xylan by acids or extraction of xylan by alkali under an atmosphere of high temperature and pressure, thereby, generating xylose. XOS production by autohydrolysis requires xylan rich biomass treatment with water under a suitable temperature-pressure combination. Both these processes lead to the production of several undesired compounds (phenolics, furfural and 5-hydroxymethyl furfural) as well conversion of a large portion of xylan into monomeric sugars [66]. Therefore, enzymatic processes for XOS have gained more interest and involves the application of hemicellulolytic, majorly xylanases and also the hemicellulose debranching enzymes. Xylanases utilized for the XOS synthesis from xylan rich biomass should have less exo-xylanolytic or β-xylosidase enzyme activities, otherwise hemicellulose would be completely hydrolyzed into its monomeric pentose sugars [67–69]. Xylan degrading enzymes can be used for xylan hydrolysis after their production and recovery or synthesized in-situ using xylanase synthesizing microbes during fermentation. Else, the enzyme can be immobilized for the synthesis of XOS [66,70]. This process is sustainable as it is environmentally friendly, utilizes renewable substrate such as agricultural residues [65,71].

### 3.4 Mannano-oligosaccharides (MOS)

MOS class of oligosaccharides derived from mannans are derived from hemicellulosic polysaccharides present in cell walls and seeds of the plants [72]. Linear mannans have β-1,4-linked mannopyranosyl moieties and glucomannan have manno- and gluco-pyranosyl moieties joined together with similar linkages. However, the side chains present in any of these two MOS are α-1,6 linked galactopyranosyl units and the MOS are, respectively, termed ‘galactomannans’ and ‘galactoglucomannans’ [73,74]. Softwoods have more proportion of gluco- and galactogluco-mannans in their hemicellulose polysaccharides, e.g. pine-biomass has more than 1/10th of the total hemicellulose in form of mannans and could serve as a good feedstock for MOS synthesis [72]. MOS provide prebiotic benefits in animals like pigs, broilers, horses, etc. These can be synthesized by the degradation of mannan by different enzymes belonging to Glycosyl hydrolases (GH) family. Endo-1,4-β-mannanase or β-1,4-D-mannan mannanohydrolase commonly termed as β-mannanase) carry out hydrolysis of the β-1,4 mannosidic bonds of mannans, galactomannan, glucomannan and galactoglucomannan and give rise to short or bioactive MOS [13].

### 3.5 Cello-oligosaccharides (COS)

Cello-oligosaccharides belong to the NDO type of oligosaccharides having a linear chain of D-glucose linked with β-1,4-glycosidic bond present with a low DP (~6), which makes them water-soluble, nondigestible to human intestine and, thereby, potential prebiotics [4]. COS improve gut microbial growth and development, especially *Lactobacillus* and *Bifidobacterium* spp [75]. Lignocellulosic biomass LCB is a source that is made by cellulose, hemicellulose, and lignin. Cellulose, the most abundant polysaccharide in nature and present in plant biomass as a major constituent, is a natural source for the synthesis of cello-oligosaccharide, which can be derived either by chemical, enzymatic, or autohydrolysis processes [10,75–78].

### 3.6 Malto- and iso-maltooligosaccharides (IMOS)

In maltooligomers, glucose moieties are joined by α-1,4-bonds and these are derived from polymeric carbohydrates (starch and glycogen) by amylolytic and pullulanases enzymes. IMOS contain α-1,6- and α-1,2-/1,3/1,4 bonded glucose and include isomaltose/triose/tetraose/pentaose, panose, nigerose, and kojibiose oligomers and are produced by starch hydrolyzing enzymes, followed by their transglycosylation by α-transglucosidase enzymes [6].

### 3.7 Other potential prebiotics

Resistant starch also remains undigested in the upper digestive system and has the potential to act as prebiotic, due to several health benefits mediated by enhanced butyrate production [79], and selective stimulation of *Firmicutes* bacterial populations [80] and fermentation by *Ruminococcus* sp., *Bifidobacterium* sp., *Bacteroides* as well as *Eubacterium* sp [21]. Branched glucans having the glycosidic linked chains can also selectively enhance some bacteria of the colon, such as *Bifidobacteria*, and therefore, possess prebiotic characters. But prebiotic potential of such glucose-derived OS or ‘polydextrose’ need to be investigated in more details, as these are still not considered under the well-recognized prebiotics like GOS, FOS, etc. [81]. Chitosan-derived oligomers are prepared from chitin polysaccharides and have N-acetyl-D- and D-glucosaminidic moieties linked through β-1,4-glycosidic bonds; whereas, another glucose derivative NDO prepared by cyclodextrin glucosyltransferase from starches include ‘cyclodextrin’ which possess cyclic α-1,4-links between glucose molecules [6]. Pectin-oligosaccharides (POS) are the depolymerized derivatives of hetero-polymeric compound pectin, and are made of α-1,4 linked acetyl/methyl substituted D-galacturonate units. The examples of POS include GOS, rhamnogalacturono-OS, oligogalacturonide, arabino-OS, arabinoxylooligosaccharide (AXOS), etc. Arabinan hydrolases, such as endo-arabinanases/galactanases, rhamnogalacturonase and arabinofuranosidases are the key POS synthesizing enzymes. Lactulose is lactose derivative connected through β-1,4 bonds and are prepared by action of β-galactosidases and glucose-isomerases enzymes; whereas, lactosucrose is a trisaccharide comprising glucose, galactose, and fructose units having β-1,4 and α-1,2-bonds. Formed by action of enzymes (levansucrase or β-fructofuranosidase) on lactose and sucrose. Isomaltulose is yet another class of α-1,6-linked oligosaccharide (sucrose isomer; alternate name ‘palatinose’), made from hexoses (fructose and glucose) [6]. The α-1,6-linked soybean-derived oligosaccharides made from soybean whey [82] include raffinose (DP 3; constituents: galactose, glucose, fructose), stachyose (DP 4; constituents: glucose, fructose, 2 galactose), and verbascose (DP 5; constituents: glucose, fructose, 3 galactoses).

## 4. LCB as a feedstock for prebiotic oligomer production

Considering the abundant supplies, renewable nature, and biological origin, lignocellulosic biomass (LCB) serves as the most suitable, sustainable and predominant source to produce biomaterials, foods, feeds and fuels. LCB is made by plants using CO_2_, water and sunlight by the process called photosynthesis. Annually, several million-ton LCB is generated in the form of agro-, industrial, forestry and municipal wastes, presenting with a global challenge of its proper disposal, which could be a major grave for the environment. Due to above-described benefits, LCB can be consider as an ideal feedstock to produce oligosaccharides [10], and other value-added products (e.g. biofuels and biochemicals) under biorefinery approach, which is considered as equivalent of petroleum refinery. A major focus of the LCB based biorefineries is on sustainability, and under this approach, various biomass components of LCB are valorized to produce multiple valuable products in a cost-effective manner, with no significant damage to the environment [83]. Many useful products can be obtained from various components of LCB, such as ethanol, oligosaccharides (COS, XOS, AXOS, MOS, etc.), sugar alcohols (xylitol, erythritol, etc.), sugar derivatives (furfural, 5-hydroxymethyl furfural, etc.), organic acids (citric acid, acetic acid, propionic acid, and lactic acid), which can be used directly or can be used as platform chemicals for converted into other biochemical products [84]. Virgin biomass, waste biomass, and energy crops are the categories in which lignocellulosic biomass can be categorized. Trees, bushes, and sand grasses are the types of virgin biomass, agricultural residue such as (rice straw, wheat straw cotton stalk, etc.), stover, and sugarcane bagasse are included in waste biomass [85].

LCB can be a good substrate for prebiotic NDOs production, because of its high content of various natural polysaccharides [86]. Biomass, once hydrolyzed by physico-chemical or enzymatic method, releases various monomeric, and oligomeric sugars, the composition and proportion of which will depend upon the biomass type, its actual composition, mechanical and physico-chemical pretreatment employed and process parameters employed during hydrolysis [10]. Structurally, LCB contains cellulose (35–50%], a homopolymer of glucose units joined to each other by β-1,4-glycosidic bond; hemicellulose (20–35%), a branched heteropolymer of 5-carbon sugars (xylose and arabinose), 6-carbon sugars (glucose, mannose, galactose, etc.), and gluco-/ galacto-uronic acid residues; and lignin (10–25%), a complex heteropolymer structure composed of phenolic components hydroxyphenyl, guaiacyl, and syringyl units. It mechanically strengthens the structure of LCB, by performing the action of ‘glue’ between cellulose and hemicellulose and covalent link with hemicellulose, thereby, providing recalcitrance to microbial and chemical damage. LCB also contains a small proportion of extractives (oils), protein and ash [8].

The major part of LCB is consisted of cellulose, which has several D-glucose as monomers arranged linearly and has a general formula of (C_6_H_10_O_5_)n (‘n’ representing the number of glucose moieties) [8].The cellulose has a very high DP (ranging from 500 to 15000), that’s why its stabilization is necessary. Hydroxyl (OH) groups in cellulose not only give stability but are also responsible for the physical and chemical behavior of cellulose through their H-bonding abilities [87]. The actual cellulose content in LCB depends upon biomass type, e.g. cellulose content of cotton fiber is approximately 90%, whereas woods contain 40–50% and dried hemp contain 57% of the total dried biomass [88,89]. Cellulose acts as a potential source of lignocellulosic-derived NDOs called cello-oligosaccharides (COS). Among various COS, cellobiose (DP value 2) has shown greater prebiotic potential as indicated by stimulation of *Bifidobacterium sp*. growth and its acceptability in humans [90]. It reaches the colon in undigested form and can be metabolized by the gut microbes via fermentation, providing health benefits to the human host by improving their metabolism, control of blood glucose as well as obesity [57,58,91]. Thus, LCB can be effectively utilized in a cost-effective and sustainable manner for the synthesis prebiotic COS which are considered safer for use as food supplements [75].

In contrast to cellulose, hemicellulose is branched, heterogeneous and highly branched polysaccharide consisting of five-carbon (pentose) sugars (xylose and arabinose) and hexoses (mannose, galactose, and glucose), and comparatively lower DP (50–200) [8]. Among various pentoses and hexoses, xylan is the principal component (60–90% wt.). Xylan, mannan and arabinan are the polymers of xylose, mannose and arabinose, respectively. Hemicellulose is also connected to other two plant components (i.e. cellulose and lignin), providing mechanical strength to plants. After cellulose, hemicelluloses are the second most abundantly available carbohydrate polymer. It is used in food, fuel, pharmaceutical, cosmetic and many other industries. Hemicellulose content also varies significantly in various plant biomass types. Hemicellulose content of birch wood is 89.3%, rice bran 46%, corn fiber 48–54%, and sugar cane bagasse 20–40% of the total biomass [92,93].

Xylobiose/-triose/-tetraose and other xylose derived oligosaccharides having DP up to 10 are the main XOS, having known prebiotic properties [94]. Moreover, in addition to XOS production, other branched XOS derivatives, such as arabino-xylooligosaccharides (AXOS) can also be produced from hemicellulose by either enzymatic, or physico-chemical methods [82]. The main constituents of AXOS are α-1,3- and α-1,5-l-arabinofuranosyl moieties and the arabinose of plant biomass is derived from arabinans, or arabino-galactans/-xylans [95]. The remaining pentose (mannose) derived component of hemicellulose, i.e. mannan, and other substituted mannan containing oligosaccharides gluco-, galacto-, or glucogalacto-mannan, can also be converted to simpler potential prebiotic mannano-oligosaccharides having lower DP values, with the help of various enzymes, including β-1,4-D-mannanases that acts upon β-1,4-bonds [96].

## 5. Process for production of lignocellulosic biomass derived prebiotic oligomers

**Figure 1.**
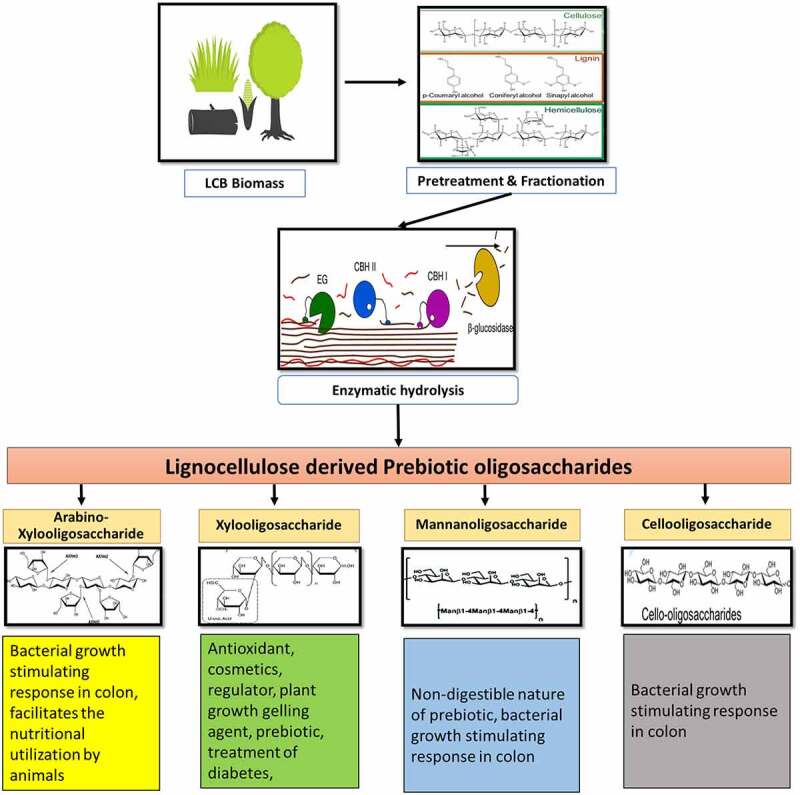
An overview of LCB-derived oligomer synthesis and their important functions.

As discussed earlier, LCB is apparently a suitable source for synthesis of prebiotic NDOs, as it is a plant derived material, with a complex structure consisting of three major structural components. However, recalcitrant nature, crystalline structure and presence of lignin, makes the conversion of LCB to value-added products a very difficult task [97]. Due to the structural complexity, it is very essential that the plant biomass is first made accessible to the enzymes or chemicals to be employed during conversion of (hemi)cellulose to various NDOs. Deconstruction of this structure is achieved by the process of pretreatment, in which, physical, chemical, or biological methods are used to reduce the complexity of the biomass. Polysaccharides, majorly hemicellulose and some cellulose, are also depolymerized during pretreatment process, releasing various monomeric, oligomeric pentose, and hexose sugars, as well as lignin or polysaccharide derived inhibitory compounds [83]. Process for production of prebiotic NDOs from the pretreated LCB feedstock involves the application of physico-chemical or biochemical route of biomass conversion, the latter being milder and highly specific with lower by-products an environmentally friendly [12]. **An overview of LCB derived oligomer synthesis and their important functions is depicted in** Figure 1.

### 5.1 Pretreatment of lignocellulosic biomass

Mechanical processing always precedes the physico-chemical or biological pretreatment and utilizes various type of milling (Willy-, ball-, roller-, or knife-mills) for trimming the biomass into powered form having smallest possible particles, so that their surface area is exposed to a greater extent for easy access of the enzymes to cellulose and hemicellulose components of the biomass. Physico-chemical pretreatment processes employ water alone or in combination with acids (sulfuric-, hydrochloric-, nitric acids, etc.), alkali (sodium or potassium hydroxide, ammonia, etc.), oxidizing agents (hydrogen peroxide, ozone, etc.) along with heat ≥ 100°C and high pressure. Steam explosion doesn’t require the addition of acid/base catalysts and results in better enzymatic digestibility by relying upon the use of pressurized (1–3.5 MPa) hot steam (~150–200°C), in which biomass is explosively decompressed to atmospheric pressure causing its rupturing and ultrastructural changes [98]. Ultrasonic pretreatment of LCB enhances the extractability of biomass components postpretreatment accompanied with significant reduction in pretreatment time with use of higher dose of ultrasonic waves. Physico-chemical pretreatment enhance the digestibility of the biomass by partial breakdown of lignin and hemicellulose, decreasing cellulose crystallinity and improving porosity and pore-size, thereby, loosening up the complex structure of biomass that results in an increased accessibility of enzymes toward the biomass components [8]. Dilute acid (DA) pretreatments of LCB specifically remove the hemicelluloses to make cellulose more amenable to cellulolytic enzymes. DA pretreatment causes the depolymerization of hemicellulose into its constituent hexose and pentose sugars by the action on ester links and glycosidic bonds of hemicellulose. It also leads to lesser production of sugar degradation products, less corrosion of the vessel, decreased wastewater generation and is more cost effective than concentrated acid pretreatment of LCB [99–101].

During alkaline pretreatment of LCB, lignin is selectively removed, while cellulose and hemicellulose components remain intact in the remaining biomass. The liquid ‘hydrolysate’ portion after the treatment, is separated from the solids by centrifugation or filtration. The solids containing the pentose and hexose polysaccharides can then be selectively hydrolyzed by (hemi)cellulolytic enzymes to produce monomeric sugars (xylose, glucose, arabinose, mannose, etc.) and LCB-derived NDOs (COS, XOS, AXOS, MOS, etc.). Dilute alkali pretreatment improves the biomass digestibility, a highly desirable feature when producing XOS through biochemical route employing enzymes [102]. Proper concentration of the base and other process parameters, like temperature, solid loading, mixing, duration, etc. play critical role in improving the xylan content. Previously, alkaline treatment of coconut husk lignocellulose with 200 g/L sodium hydroxide for 1 h resulted in >90% removal of xylan and enhanced XOS production via enzymatic hydrolysis [103]. Organosolv pretreatment, involving the use of organic solvents alone or in combination with catalyst, is increasingly being used now days due to solubilization and recovery of pure lignin for further industrial exploitation. Karnaouri et al. [1], explored its applications in production of COS from Birchwood.

Biological pretreatment of LCB employs whole microbial cells or their enzymes such as manganese peroxidase, laccases, etc. (either in free or immobilized form) for breakdown or removal of lignin. Biological pretreatment is the most environmentally friendly method of pretreatment due to low chemical, cost and energy inputs and lesser waste outputs. White-rot fungi are the predominant type of biological agents used and the examples include, *Phanerochaete* sp., *Pycnoporous* sp., *Ceriporiopsis* sp., and *Ganoderma* sp. However, the greatest difficulty in using this method is the extremely slow pace of delignification [104], which makes it industrially less favorable in comparison to physico-chemical pretreatment methods.

Once, the LCB feedstock has been subjected to the physico-chemical or biological pretreatment, it can be efficiently converted to the desired type of prebiotic oligosaccharides via physico-chemical or biochemical conversion.

### 5.2 Physico-chemical conversion route for production of LCB-derived prebiotics

Physico-chemical methods used to obtain prebiotic oligomers from LCB include thermo-chemical and autohydrolysis processes, of which the former requires the use of some sort of catalysts, whereas, the latter doesn’t [65]. Thermochemical processes involve hydrolysis or selective removal of LCB components using acid or alkali, along with application of heat and high pressure. Thus, thermochemical conversion route leads to better efficiency of conversion of biomass components into their respective oligosaccharides; however, this process also leads to production of unwanted compounds such as phenolics and furans, release of monomeric sugars, as well as sugar loss [105]. Recently, for production of XOS/COS, sugarcane bagasse was treated with sulfuric acid in presence of heat and pressure to obtain LDOs concentration of 14 g/L [106]. For XOS and other LDOs production, the autoclave and parr reactors have been commonly employed for lab-scale studies, because it can easily control the heating and cooling curve thus the effect of set temperature can be accurately determined by these reactors [12,13]. Acid and bases are used to hydrolyze LCB by using of different physical factors like temperature, pressure and time that determine the degree of polymerization of oligosaccharides. Dilute sulfuric acid pretreatment with (0.1–0.5 M) at low temperature (<160°C) and at high temperature (>160°C) is commonly used for XOS production [107,108]. NaOH, KOH, Ca[OH]_2_ and combination of these alkalies are used for the extraction of Xylan from LCB then xylan can be converted into XOS by using of xylanase enzymes, the enzymatic process may be slower process as compared to autohydrolysis [60].

Another type of physico-chemical method of LCB to oligosaccharide conversion is the autohydrolysis process in which high temperature (between 150 and 240°C) and pressure (up to 10 Mpa) are applied to the biomass in presence of appropriate water content [101]. Alternate names for this process are ‘hot water pretreatment’ and ‘hydrothermal treatment.’ Autohydrolysis provide better and faster biomass conversion rates of hemicellulosic polysaccharides into pentosans and there is no requirement of the catalyst in this treatment process. This process has been used applied for quick and simpler production of XOS from hemicellulosic biomass of Oil palm. Autohydrolysis treatment of oil palm biomass was applied at 121°C for 1 h prior to xylanase treatment, with an enzyme dose of 80 U/g substrate, to produce more than 17% XOS [109]. Recently, Jang et al. [110] employed autohydrolysis of sweet sorghum bagasse (SSB) biomass at 180°C for 20 min and achieved high yield of XOS (67.6%) having lower DP values. Acetylation of biomass and autoionization of water on high temperature and pressure starts the hydrolysis process. Therefore, when this process is applied, most of the hemicellulose are removed from the biomass remain dissolved in the liquid hydrolyzate. The yield of oligosaccharide could be increased by application of optimum time-temperature combinations [111,112]. Despite some advantages, autohydrolysis treatment suffers from the demerits similar to that of acid/alkali based thermo-chemical conversion, such as higher formation of monomers and degradation products (acetic and formic acids, furans, phenolics, etc.) [66,68,105]. Due to production of various harmful chemicals, the produced NDOs need to be purified to a higher level, which also increases the input costs of prebiotic production. For example, centrifugal partition chromatographic analysis was applied for autohydrolysis treated Pinewood biomass for separation of mono- and oligomers from phenolics and furans [113]. Moreover, autohydrolysis has been used for AXOS production from LC biomass of Ryegrass [82]. Birchwood xylan and sugarcane bagasse were also recently treated with autohydrolysis for the production of XOS (DP 2–5) and COS/XOS, respectively [106,114]. In the latter study, autohydrolysis was found to significantly enhance the yield of oligosaccharides than monomeric sugars, in contrast to sulfuric acid treatment. In the recent past, application of subcritical water–carbon dioxide treatment has also been described as a suitable process for NDOs production from xylan rich biomass [93].

### 5.3 Biochemical conversion route for production of LCB-derived prebiotics

Controlled enzymatic hydrolysis of cellulose is believed to be mild and environmentally friendly in comparison to the physico-chemical processes, results in high specificity and generates lesser waste-effluents, as well as lesser sugar loss in form of monomers and toxic by-products. Biochemical conversion of LCB feedstock into prebiotic oligosaccharides require the application of lignocellulolytic enzymes for the breakdown of plant polysaccharides into low DP (2–10) oligomers. The structure of LCB is complex, hemicellulose being more complicated than cellulose, therefore, requiring different set of enzymes with different specialties for generating variety of LDOs upon enzymatic hydrolysis. Production of XOS require application of xylanases, that of AXOS require the debranching enzymes, whereas cellulose hydrolysis for COS production requires the action of cellulases [68]. Various aspects of production of lignocellulose-derived oligosaccharides via enzymatic hydrolysis are summarized in Table 2.

**Table 2.** Production of lignocellulose-derived oligosaccharides via enzymatic hydrolysis

LDO type	Feedstock	Enzyme	Hydrolysis conditions	Oligomer yield	Characterization method	References
**XOS**	*Populas tomentosa*	Crude xylanase	U-25 U/g subs pH- 5.4 Temp- 50°C	DP 2–4 Yield- 3.95 mg/mL of the hydrolyzate	-	[115]
**XOS**	MPSA (Dilute sulfuric acid-pretreated sugarcane)	α-L-arabinofuranosidase, endo-1,4-xylanase	U-25 mg/g subs Temp- 50°C Time- 48 h	DP 2–6 Yield-261.23 mg/L	HPLC-PAD	[148]
	MPIL (Ionic liquid-pretreated sugarcane)	α-L-arabinofuranosidase, endo-1,4-xylanase	U-20 mg/g subs Temp- 50°C Time- 48 h	DP 2–6 Yield-249.54 mg/L		
**XOS**	Red alga dulse (*Palmaria* sp.)	Commercial hemicellulase amano 90	U- 54 U Solid loading- 10 mg/mL pH 4.5, Temp- 50°C Time- 24 h	DP 2–3 Xylobiose- 1.1 mg/mL Xylotriose- 0.3 mg/mL	HPLC	[116]
**XOS**	Corncob	Acetic acid hydrolysis and enzymatic hydrolysis	U − 15 U /g cell solid loading- 10% pH- 4.8 Temp- 50°C Time- 72 h	DP 2–6 Yield-139.8 g/kg	HPAEC-PAD analysis	[164]
**XOS**	Sugarcane bagasse	Xylanase (A. *fumigatus* M51)	FPU – 500 U /g substrate pH- 5.0 Temp- 50°C Time- 72 h	DP 2–3 Yield- 1.04 mg/mL	HPLC	[117]
			(*T. reesei* CCT 2768) U- 500 U /g substrate pH- 5.0 Temp- 50°C Time- 72 h	DP 2–3 Yield- 0.88 mg/mL		
**XOS**	Corn stalk (CS)	Xylanase	U- 3.4 × 10^4^ IU/g pH- 5.0 Temp- 50°C Time- 24 h	DP 2–7 Yield- 77.4%	HPLC; purification by activated carbon-ethanol method	[158]
**XOS**	Beechwood xylan (BX) Rye arabinoxylan (RX)	Two-domain GH10 xylanase from *Jonesia denitrificans*	U- 84 U/mg (BX) U- 65 Umg (RX) pH- 2-10 Temp- 35°C Time- 6 h	DP 2–6 Yield- 47.67% (BX) & 26.01 (RX)	HPAEC	[118]
**XOS**	*Sehima nervosum* grass	Commercial xylanase	U- 17.41 U pH- 5.03 Temp- 45.19°C, Time- 10.11 h	DP 2 Yield- 110 g/kg Xylan	HPLC with RI detector using ZORBAX carbohydrate column	[119]
			U- 13.20 U pH- 5.11 Temp- 40.33°C Time- 16.55 h	DP 3 Yield- 11 g/100 g Xylan		
**COS**	Forest waste	Commercial Celluclast® with beta-glucosidase inhibitor	U- 25 mg/g pH- 7.0 Temp- 50°C Time- 24 h Conduritol B epoxide-1.98 mM	Yield- 128 mg/ g biomass	Purification by Nanofiltration	[1]
**COS**	*Ciona intestinalis*	Controlled hydrolysis with a Combination of Cellobiohydrolase I (CBH1) and endoglucanase EG5	U- 20 mg/g solids pH- 5.0 TEMP- 50°C, Time- 24 h	DP 2 conversion yield – 49.7 wt. %.	HPLC	[120]
**COS**	Sugarcane straw	Enzyme cocktail (endoglucanases CaCel and CcCel9m, the LPMO TrCel61A, the CDH NcCDHIIa, with lactose and copper as additives)	pH- 5.0 TEMP- 50°C, Time- 48 h	DP 2–5 Yield- 60.49 mg/L	HPAEC-PAD with a Dionex ICS-5000 ion chromatograph	[127]
**AXOS**	Brewers’ spent grain (BSG)	Commercial enzymes	Substrate loading- 20 g/L U- 2 /mL pH- 4.5 Temp- 40°C, Time- 12 h	DP 2–5 Yield- 52.0 mg/g	TLC	[121]
		Direct fermentation using *Trichoderma* species	U- 2 U/mL. pH- 7.0 TEMP- 30°C, Time- 72 h	DP 2–5 Yield- 38.3 mg/g		
**AXOS**	Hydrothermally Pretreated ryegrass	*Cellovibrio mixtus* endo-1,4-β-xylanase (GH10, Megazyme)	U- 70 U/g pH- 5.0 TEMP-40°C Time- 24 h	DP 2–4 Yield- 5.5 g/L	HPLC	[82]
		*Thermotoga maritima* endo-1,4-β-xylanase (GH10 Megazyme)	U- 70 U/g pH- 5.0 Temp-40°C Time- 24 h	DP 2–4 Yield-4.8 g/L		
**MOS**	Locust bean gum	β-mannanase from *Bacillus pumilus* GBSW19	U- 10 U/mg pH- Temp- 50°C Time- 24 h	DP 2–6, Yield- 1.19 mg/mL		[122]
**MOS**	Chinese honey locust (*Gleditsia* *sinensis*)	Commercial mannanase	U- 8.1 U/g pH- 4.0 Temp- 57.4°C Time- 34.1 h	DP 1–5 Yield-29.1 g/L		[123]

Cellulases are complex endoglucanases (EG), exoglucanases and β-glucosidases. Similarly, xylanases possess three major enzymes, exo-/endoxylanase and β-xylosidase; however, due to heterogeneity of hemicellulose polysaccharide, many other enzymes, including various de-branching enzymes, are needed in addition to xylanases for hemicellulose degradation. Examples of accessory hemicellulolytic include arabinofuranosidase, acetylxylanesterase, mannosidases, arabinosidases, and feruloyl esterase, which show synergy with core (hemi)cellulolytic enzymes and act in a sequential manner to bring about the complete depolymerization of the plant polysaccharides into their respective monomeric sugars in a processive manner, which is highly desired during bioethanol production [8,124]. As complete hydrolysis of lignocellulose components is not required during enzymatic production of LDOs, xylanases or cellulases with comparatively lower exo-xylanase/cellulase or β-xylosidase/glucosidase activities and higher endo-glucanase/xylanase activities are preferred for XOS/COS production from LCB [67–70]. Many process parameters influence the enzymatic LDOs synthesis, such as incubation time, temperature and agitation speed, enzyme dose, pH of the reaction mix, in addition to the type and amount of plant polysaccharides and the efficacy of the enzyme [103].

Hydrolytic enzymes endoxylanase, β-xylosidase, and debranching enzymes acetyl esterase are needed for conversion of xylan into XOS and the reaction also leads to the formation of xylose. XOS synthesis with a yield of 53% after 1 d incubation during enzymatic saccharification of alkali-pretreated cotton stalk with xylanase was reported recently [125]. XOS having DP ≤5 was successfully harvested by ultrafiltration using 10 kDa membrane. Similarly, dilute ammonia-pretreated corn stover resulted in 35% yield of LDOs (glucurono-XOS and xyloglucan OS) with DP ≤5 [126].

Likewise, conversion of mannans to MOS, cellulose to COS, arabinoxylans to AXOS, arabinans to AOS, galactans to GOS and pectin to POS require specific pair of enzyme activities, however, such polymeric sugars are present in a relatively lower proportions in the hemicellulose, e.g. the highest galactan content of LCB is only 25 g/kg dry biomass of Radiata Pine [12].

For COS production, apart from the usual cellulolytic enzymes acting on the cellulose chain in a processive manner, some accessory enzymes such as lytic polysaccharide monooxygenases (LMPOs) and cellobiodehydrogenase (CDH) are also needed to oxidatively cleave random sites in the cellulose chains [127–131]. LPMO act in synergy with hydrolytic enzymes providing new regions for cellulase action due to their ability to oxidize glycosidic bonds [132–135]. LPMOs have been shown to generate new ends in crystalline regions of cellulose chains, thereby, enhancing the proportion of amorphous regions in the biomass. Apart from LPMOs, another potential accessory enzyme CDH have also been described to oxidize cellobiose. CDHs transfer electrons to LPMOs, thereby, assisting in the oxidative cleavage of cellulose [136–139]. Some EGs have been reported to possess both endo- and exo-activities bringing about the processive breakdown of larger OS from noncrystalline region of cellulosic polysaccharides due to presence of catalytic domain of GH family 9 and a carbohydrate-binding module (CBM) [140].

Cellobiose production from LCB substrates, spruce and birch, has been previously attempted by employing CBH7 and EG5, which are members of GH family 7 and 5, respectively [9]. These two enzymes act processively on cellulose fibrils, generating soluble oligosaccharides from chain terminals [141,142]. Karnaouri et al. [75], studied application of prokaryotic and fungal EGs of GH family 9, 6, and 48 in LDO production, and reported cellobiose as the major COS, exhibiting prebiotic property of promoting *Lactobacilli* sp. and *Bifidobacteria* sp. growth.

### 5.4. Recovery, purification and characterization of LCB-derived prebiotics

Recovery and purification of prebiotic NDOs after their synthesis via physico-chemical or biochemical conversion route requires the application of various downstream processing steps, including precipitation, chromatography, extraction using various solvents, ion-exchange, evaporation, and membrane-based processes, Membrane separations processes (including, nano-/ultrafiltration). The recovery process is laborious requiring multiple steps and may become costlier, if the production method generates more by-products or monomeric sugars. Therefore, for overall cost reduction, it is necessary to choose appropriate method for generation of specific types of prebiotic oligosaccharides from the LCB substrate. Knowledge of physical, chemical and other properties of the potential prebiotic, waste generation, stability, impurities, is essential for deciding the recovery process [103,143].

Characterization of the produced oligosaccharides’ structure is of utmost importance for determining the exact functional properties. Nuclear magnetic resonance (NMR) spectrometry and mass spectrometry (including MALDI-MS, MALDI-TOFMS ESI-MS, ESI-MS, ESI-MS/MS, Fourier transform ion cyclotron resonance (FT-ICR) MS, positive tandem-MS with ESI, as well as IR spectroscopy, have been most prominently used recent methods for NDO characterization and have been reviewed in detail [143].

### 6. Recent advancements in controlled enzymatic hydrolysis of lignocellulosic biomass for prebiotic polysaccharide production

Recently, awareness for sustainable production of high-value chemicals and products including NDOs from cheaper and renewable resources has increased enormously due to increasing interest in bioeconomy and biorefineries. Advancement in technology has made the discovery and application of superior enzyme preparations for the production biomass derived NDOs a lot easier. Obviously, enzymatic production of prebiotic LDOs is a preferable method due to several advantages described in the previous section. However, choice of the proper strategy for enzymatic hydrolysis LCB is of utmost importance during high-yield synthesis of specific oligosaccharides in a proportion higher than the monomeric sugars. The most useful approaches that are recently reported for high yield production of LDOs via controlled enzymatic conversion of LCB are application of custom-made cocktails of enzymes [75], stepwise hydrolysis of LCB [15], use of enzyme inhibitors [16], application of heterologous expressed lignocellulolytic enzymes [9] and enzyme reaction engineering by modifying temperature, pH and other hydrolysis conditions [1]. Some of the prominent strategies employed for the enhanced enzymatic conversion of LCB into prebiotic oligosaccharides are summarized in Table 3 and discussed below.

**Table 3.** Recent research advancements in enzyme and microbial technology for lignocellulose-derived oligosaccharides production

S. no.	Targeted products	Strategy for improving enzymatic synthesis of oligosaccharides	Major highlights of research	References
1.	XOS (majorly xylobiose and xylotriose) and glucose	Synergistic custom-made enzyme cocktail using in-house recombinant enzyme	EH of sugarcane straw xylan (72.56% xylan conversion) by heterologous endoxylanase of *Cryptococcus flavescens*, in *Pichia pastoris* GS115 in synergism with commercial arabinofuranosidase XOS production optimization by statistical design (CCRD) Remaining glucan rich biomass hydrolyzed to produce glucose	[149]
2.	XOS (DP 2–6, majorly xylobiose)	Synergistic custom-made commercial enzyme cocktail	Commercial hemicellulases (endoxylanase and arabinofuranosidase (GH51)) cocktail composition optimized for production of XOS (DP 2–6) by CCRD Ionic liquid pretreatment of sugarcane bagasse and straw mixture lowered enzyme dose up to 20% due to more delignification than dilute sulfuric acid pretreatment	[148]
3.	XOS (majorly xylobiose, xylotriose, and xylose)	Fed-batch mode of enzyme hydrolysis	Maize straw EH under fed-batch mode with 2% solid loading and xylanolytic enzyme at a dose of 12 U/g for 7 h resulted XOS yield of 0.67 g/g XOS had antioxidant activity under in-vitro conditions, with inhibition of HepG2 cells, suitable for use as antioxidant and anti-cancer ingredient for food or pharma applications	[152]
4.	XOS	Process improvisation by adopting integrated approach for combined autohydrolysis, nanofiltration and enzymatic hydrolysis	Removal of by-products and monomers via nanofiltration with discontinuous diafiltration High recovery of XOS (84%) and xylan (87%) by xylanase mediated EH of autohydrolysates of biomass EH increased yield to 96–98%, with final XOS conversion of 41%	[166]
5.	XOS (majorly xylobiose), xylose, and butanol	Biorefinery approach	Coproduction of XOS & butanol from steam explosion (SE) pretreated *Eucalyptus grandis* biomass Effect of temperature on selective production of XOS using a pre-pilot SE reactor with 50% xylan conversion and 80% glucan saccharification under higher solid-loading Ion-exchange and resin treatment of XOS-rich hydrolyzate improved XOS recovery Enzymatic hydrolyzates used for butanol production by *Clostridium beijerinckii*	[98]
6.	XOS (major-DP6, 3, and 4)	Custom-made thermophilic enzyme	Thermophilic GH11 endo-b-1,4-xylanase obtained from a metagenomic library from sugarcane bagasse having optimal temperature of 80°C and pH 6 CBM trimming (X11C) and Pro71Thr mutation by random mutagenesis increased hydrolytic efficacy of enzyme by ~16× and 6.5× while of wild type, resp. Best XOS yield of 5.5 g/mg enzyme (~3.7× than wild) & >800 mg/g xylan	[163]
7.	XOS (DP 2–6, major- xylobiose and xylotriose and very low xylose)	Custom-made enzyme having catalytic and binding domains	Biochemical characterization of recombinant 2-domain (GH10 and CBM2 domains) xylanase of *J. denitrificans* with sp. activity 84 and 65 U/mg protein on beechwood glucuronoxylan and rye arabinoxylan, resp. EH of beechwood and rye xylan with 48 & 26% efficiency Homology modeling suggested compression of +2 subsite as main reason for lower monomer yield	[118]
8.	COS and cellobiose	Synergistic custom-made enzyme cocktail	Cellobiohydrolases (CBHI and II) and endoglucanases (GH 5, 6, and 9) of *Thermothelomyces thermophila* for synergistic production of cellobiose from organosolv pretreated spruce and birch	[9]
9.	AXOS (DP 2–6)	Direct microbial fermentation of LCB	Brewers’ spent grain directly fermented by genetically modified *Bacillus subtilis* with xylanase gene *xyn2* from *T. reesei* to improve the AXOS production with yield of 54 mg/g), with 33% increase. Single-step fermentation with *B. subtilis* outcompeted EH method	[168]
11.	XOS (major- xylobiose, followed by xylotriose)	Custom-made enzyme cocktail	Kenaf (*Hibiscus cannabinus*) stems exposed to EH by xylanase alone or by cocktail of xylanase & L-arabinofuranosidase (40:1 ratio), resulting in hemicellulose conversion (95%) & XOS production (mg/g) of 351, Xylobiose 135 & xylotriose 102 at 40°C, pH 4 and incubation period of 2 d	[69]
12	XOS (xylobiose to xylohexose) and fermentable sugars	Biorefinery approach for coproduction or oligosaccharides and fermentable sugars	Prehydrolysis of corncob by acetic acid hydrolysis (46%) followed by EH (91% conversion) resulting in ~140 g XOS, 328 g glucose, 25 g cellobiose, and 148 g xylose from 1 kg initial biomass	[164]
13	XOS	Custom-made enzyme cocktail	Sugarcane straw & coffee husk arabinoxylan subjected to EH by optimal mixture of commercial endoxylanase (GH11), arabinofuranosidase (GH51), & feruloyl esterase (CE1), resulting in 10.23 and 8.45 g/L XOS production	[150]
14.	XOS (majorly-xylobiose/-triose/-tetraose)	Crude enzyme lacking β-xylosidase	EH mediated conversion of ammonia pretreated sugarcane bagasse xylan to XOS (>99%) with no monomeric pentose production when using crude xylanase *Bacillus subtilis* lacking β-xylosidase activity XOS characterization by MALDI-TOF-MS and HPLC revealed DP 2–4 as major products & NMR characterization showed arabinosyl & glucuronyl substitution in 32% XOS XOS stimulated *Bifidobacteria* growth	[153]
15.	XOS	Custom-made recombinant enzyme	Recombinant thermostable xylanases of *M. thermophilus* heterologous expressed in *P. pastoris* for EH of beechwood xylan at pH 6–6.5 and 65°C, supporting growth of *Lactobacillus* sp.	[160]
16.	XOs (xylobiose, xylose & xylotriose as major products)	Immobilized, custom-made recombinant enzyme	Recombinant immobilized endoxylanase of *B. subtilis*, increased thermostability at 56°C and pH 5.5) produced XOS (DP 2–4) using soluble and solid Birchwood xylan, with 20% xylan conversion within 3 h, without monomer accumulation Enzyme recyclability up to 10 cycles of EH	[167]
17.	XOS	Fine-tuned enzymatic hydrolysis using thermostable enzyme	Thermostable endoxylanase of *Bacillus velezensis* purified to >5-fold for EH of sugarcane bagasse for XOS production, supporting *Bifidobacterium* growth	[144]
18.	GXOS	Fine-tuned enzymatic hydrolysis using glucuronosyl requiring enzyme	Alkali extracted glucuronoarabinoxylan of Quinoa stalks subjected to EH by glucuronosyl-requiring GH30 enzyme for production of glucuronosylated-XOs (GXOs)	[145]
19.	COS (cellobiose, cellotriose, and cellotetraose)	Custom-made enzyme cocktail/Use of enzyme inhibitor	Four EGs and 2 BGL purified from digestive fluids of the sea hare (*Aplysia kurodai*) and found to act synergistically on cellulose for COS production Filter paper hydrolyzed by cellulase to COS BGL inhibitor D-glucono-1,5-lactone for optimal COS recovery	[16]
20.	COS (majorly cellobiose)	Stepwise hydrolysis	Multistage separation of EH filtrate using vacuum-filtration and resuspending retentate for hydrolysis of leftover biomass by the available enzyme Higher (approx. 45%) cellobiose production in multistep hydrolysis process versus that in uninterrupted process caused by β-glucosidase loss during filtration and lesser product inhibition	[15]
21.	XOS (majorly xylobiose and minor xylotriose/ xylotetraose with arabinose/glucuronic acid substitution) and monomers	Stepwise hydrolysis	Stepwise EH of alkaline oxidation (AO) treated bagasse with xylanase and cellulase to coproduce XOS (1.78 g/L), and monomer (~92% cellulose conversion) synthesis	[151]
22.	COS (majorly cellobiose)	Fine-tuned enzyme cocktail by use of enzyme inhibitor and enzyme reaction engineering	Fine-tuning commercial cellulolytic cocktail for enhanced cellobiose production via enzyme reaction engineering by use of optimal pH, multistep EH, β-glucosidase inhibitor (conduritol-B-epoxide) Cellobiose-enriched COS production from organosolv pretreated Birchwood Enhanced COS recovery by ultra- and nanofiltration	[1]
23.	XOS (DP 2–6), bioethanol, and lignin	Custom-made enzyme cocktail under biorefinery approach	Steam explosion pretreated barley straw subjected to EH by cocktail of endoxylanase and accessory enzymes arabinofuranosidase, feruloyl-/acetylxylan-esterases and produced XOS at 130 g/kg substrate Other biorefinery products were bioethanol 126 g/kg and lignin-rich residual biomass having heating value of 23.4 MJ/kg	[165]
24.	XOS (xylobiose and xylotriose)	Protein engineering (molecular evolution approach)	Improved catalytic performance of GH11 xylanase XynLC9 of *B. subtilis* via its mutation by site saturation and iterative mutagenesis at N-terminal residues 5-YWQN-8 in XynLC9 Mutants had 2.6× and 1.8× more catalytic activity, with better thermostability, lower substrate affinity, higher turnover rate (*k_cat_*), 1.6× more XOS production from corncob-extracted xylan	[158]
25.	XOS (xylobiose, xylotriose, and xylotetraose)	Protein engineering (rational approach for thermostability improvement)	Recombinant thermophilic GH11 xylanase gene Tlxyn11B of *Talaromyces leycettanus* with high catalytic efficiency expressed in *Pichia pastoris* with greatly improved activity (8259 U/mg protein) and pH stability (pH 1–10.5) EH of beechwood xylan by recombinant enzyme released XOS with DP 2–4 Structure-based rational method of mutation of N-terminus of enzyme improved for higher thermostability at 67°C	[159]
26.	XOS (xylobiose-xylopentaose)	Protein engineering (laboratory evolution via DNA shuffling)	Endoxylanase with high specific activity, thermostability, and broad pH adaptability Mutant library made for GH11 endoxylanase by DNA shuffling of catalytic domain of parental strains *Bacillus amyloliquefaciens* xylanase A (BaxA) and *Thermomonospora fusca* TF xylanase A (TfxA). Best mutants (DS153, DS241, and DS428) had higher activity 4.5-, 4.6-, and 3.9-fold than recombinant reBaxA, optimum pH 6, 7, and 6, respectively Three mutants have identical hydrolytic function as reBaxA, which released xylobiose-xylopentaose from oat spelt, Birchwood, and beechwood xylan Distal single residue substitution improved catalytic efficiency of xylanase at atomic level	[161]
27.	XOS (majorly xylobiose)	Protein engineering (error-prone polymerase chain reaction and DNA shuffling)	Endo-xylanase of *Thermobifida fusca* improved via error-prone polymerase chain reaction and DNA shuffling G4SM1 mutant (S62T, S144C, N198D, and A217V) with highest activity, wide pH stability (5–9) and 8.5× thermal stability at 70°C heterologous expressed in *P. pastoris* under GAP promoter resulting in 2.12× better sp. activity, due to 2-amino-acid amino acid changes at catalytic domain resulted better XOS yield from xylan	[162]
28	COS	Metagenomics	CelM encoding EG cloned from thermal spring, having high thermal, alcohol and saline tolerance EG was active at 30–95°C, working most efficiently at 80°C COS production from amorphous cellulose	[146]
29	XOS (majorly xylobiose, xylotriose, xylotetraose, xylopentaose, and xylohexaose)	Metagenomics	Novel xylanase (XynM1) isolated from extremophilic aquatic habitat XynM1 worked efficiently at 80°C, and pH 7.0, with high temperature, pH and salt stability High XOS recovery from XynM1 hydrolyzed beechwood xylan	[147]

### 6.1 Tailor-made synergistic enzyme cocktails

Preparing tailor-made enzyme cocktail or tuning the performance of available enzymes is highly advantageous, to match the repertoire of enzymes actually needed to bring about the efficient conversion of structurally different biomasses. For tailoring or customization of lignocellulosic enzymes cocktail, the selection of different biocatalysts that can act in synergistically is very important, because a single enzyme is not well enough to valorize the complex structure of lignocellulose. Use of recombinant approaches could help in preparing customized enzymes with desired characteristics. Recently, Karnaouri et al. [75], made an effective enzyme cocktail to valorize the forest waste for oligosaccharide, by combination of four different cellulases (EG5/7, CBH6/7) and one accessory enzyme lytic polysaccharide monooxygenase. Different recombinant enzymes having different characteristics were screened to determine their synergy on different polysaccharides like avicel, carboxymethylcellulose and phosphoric acid swollen cellulose. After screening of enzymes, they concluded that *Ct*CBH5A, *Pa*CBH6A, and *Cc*Cel9A effectively produced cellobiose. Although *Ct*Cbh5A, *Ct*Cel9B, *Pa*Cbh6A, *Cc*Cel9W, *Cc*Cel9M, *Cc*Cel9J, and *Cc*Cel9Q could produce not only C2 products, but also C3 and C5 sugars. *Ct*Cbh5A, *Ct*Cel9B, *Pa*Cbh6A, *CcCel*9W, and *Cc*Cel9A, had exo-activity and released C2 and C3, but not C4 and C5 products. When *Ct*Cbh5A, *Pa*Cbh6A, *Cc*Cel9M, and *Cc*Cel9A were applied in different type. With optimal enzyme combinations, birch and spruce cellulose could be hydrolyzed to cellobiose with 22 and 19% conversion, respectively. The scale-up part was also done and final oligosaccharide mixture had > 90% cellobiose.

In very recent past, synergistic custom-made enzyme cocktails have also been used for the synthesis of XOS from sugarcane biomass by use of thermostable recombinant endoxylanase of *Cryptococcus flavescens* in synergic association with commercial arabinofuranosidase (GH 51) [148], commercial hemicellulase cocktail of endo-1,4-xylanase, and α-L-arabinofuranosidase (GH51) [149], and optimal mixture of three commercial enzymes endoxylanase (GH11), α-L-arabinofuranosidase (GH51), & feruloyl esterase (CE1) [150]. Similarly, for prebiotic XOS production, Henaf (*Hibiscus cannabinus*) biomass was saccharified by cocktail of xylanase and L-arabinofuranosidase in an optimized ratio of 40:1, resulting in maximum hemicellulose conversion (95%) and yields (mg/g) of total XOS of 351, Xylobiose 135 and xylotriose 102 [69]. Karnaouri et al. [9], tailor-made a synergistic enzyme cocktail comprising of cellobiohydrolases (CBHI & II) and endoglucanases (GH 5, 6, and 9) of *Thermothelomyces thermophila* for synergistic production of COS (majorly cellobiose) pretreated spruce and birch biomasses.

### 6.2 Enzyme reaction engineering

Under this strategy, possible approaches are: stepwise or multiple-step hydrolysis of biomass, alterations in various variables known to affect enzyme saccharification, temperature, pH, etc. Stepwise hydrolysis of alkaline oxidation (AO) pretreated bagasse was recently performed by Li et al. [151], using xylanolytic and cellulolytic enzymes for coproducing XOS (~1.8 g/L) and monomers (~92% cellulose conversion). XOS majorly contained xylobiose, with small quantities of xylose and substituted xylotriose and xylotetraose. Karnaouri and coworkers [1], implemented the strategy of multiple-step hydrolysis of Birch biomass with buffer exchange method, which didn’t allow the accumulation of monomeric sugars and lead to better and faster enzyme activity for achieving higher LDO titers. In the same study, enzymatic hydrolysis conditions were also modified and the determined optimal time, temperature and pH conditions were 24 h, 50°C, and 7.0, respectively, for maximal enzymatic synthesis of XOS. After fine-tuning of enzymatic hydrolysis and enzyme-reaction engineering with a buffer exchange at 8 h, and product recovery by ultra/nano-filtration, production of oligomers resulted in high C2 to C1 ratio of 37.4:1.

Changing the enzyme reaction process parameters (time, temperature, pH, etc.) can lead to fine tuning of the performance of the desired enzymatic activities, while inhibiting the undesired ones. Multistep hydrolysis with intermittent recovery of the sugars from the hydrolyzed biomass is also a very useful yet simplistic approach which allows for higher recovery of OS than the monomeric sugars. This approach has recently resulted in enhanced COS production with cellobiose as major product (45% hydrolysis) by multistage separation of enzymatic hydrolyzate using vacuum-filtration and resuspension of retentate for hydrolysis of leftover biomass by the available enzyme. High OS yield was reportedly due to β-glucosidase loss during filtration and lesser product inhibition [15].

Moreover, an innovative strategy of fed-batch mode of enzyme hydrolysis with xylanolytic enzyme has also been adopted for increasing antioxidant and anticancer activity possessing XOS production yield to 0.67 g/g maize straw [152].

### 6.3 Inhibition of specific enzyme activities

Most of the biomass degrading enzymes are processive, acting sequentially for depolymerizing biomass into their constituent monosaccharides. The processivity of biomass degrading enzymes is a desirable feature when fermentable sugars production is a top priority, especially for bioethanol applications [8,124], but it is highly undesired for oligosaccharide synthesis. Therefore, for LDO production, those enzymes are preferred that have lower exo-hydrolytic or β-xylosidase/glucosidase activities, but higher endo-hydrolytic activities [67–69]. For example, a crude xylanase of *Bacillus subtilis* lacking β-xylosidase activity was employed successfully for superior (>99%) conversion of sugarcane bagasse xylan to XOS with no monomeric pentose production [153].

Alternatively, specific enzyme activities of the employed cocktail of enzymes, can be selectively inhibited to decrease the conversion of oligomers into monomers, thereby, achieving better oligomer yields. Recently, conduritol B epoxide (structural analogue) was employed (concentration of 1.98 mM) as beta-glucosidase inhibitor that resulted in the controlled enzymatic hydrolysis of organosolv pretreated Birch using a commercial enzyme Celluclast, with cellobiose yield of 141 kg/g substrate [1]. The selection of commercial enzyme for this study was on basis of low performance of beta-glucosidase, needed to prepare the enzyme cocktail for the production of oligosaccharides. In a similar approach Tsuji et al. [16], achieved better COS production with cellobiose, cellotriose, and cellotetraose as major products, by using D-glucono-1,5-lactone as inhibitor of beta-glucosidase during hydrolysis of cellulosic substrate.

### 6.4 Heterologous expression and protein engineering approaches

In the current era cellulolytic enzymes are very important catalyst for the deconstruction of lignocellulosic waste, for paper, pharmaceutical, bioenergy, textile, and food industries. Therefore, innovative irrational (directed evolution, DE), rational and semirational design approaches of protein engineering have been looked upon by researchers to improve enzyme’s quality, turnover number, stability, etc. in adapting to various application conditions and structure-function relationship [154–157]. Former two approaches are commonly employed for enzyme improvements to suit commercial applications. DE speeds up the evolution of proteins and delivers robust and rapid results, but making large libraries of mutants makes the screening part laborious, whereas, rational-design approach makes use of the already available knowledge for site-directed mutagenesis via computer-aided designs, with less laborious and faster analysis of mutant sequences. Molecular evolution-based protein engineering approach has been applied for enhanced catalytic performance, thermostability, turnover rate (*k_cat_*), lower substrate affinity, 1.6× more XOS (xylobiose and xylotriose) production from corncob-extracted xylan [158]. In another study, rational approach for thermo-alkali-stability improvement of recombinant thermophilic GH11 xylanase of *Talaromyces leycettanus* by its mutation at N-terminus region and expression in *Pichia pastoris* EH was applied for better release of XOS with DP 2–4 from beechwood xylan [159].

Recombinant thermostable xylanases of *M. thermophilus* was heterologous expressed in *P. pastoris* for enhanced saccharification of beechwood xylan for XOS synthesis at high (65°C) temperature [160]. Liu et al. [161], improved catalytic efficiency of xylanase at atomic level by laboratory evolution via DNA shuffling for distal single residue substitution of catalytic domain of GH11 endoxylanase of *Bacillus amyloliquefaciens* and *Thermomonospora fusca* for enhanced XOS (DP 2–5). In a different approach, error-prone polymerase chain reaction and DNA shuffling of xylanase of *Thermobifida fusca* was performed by Wang et al. [162], followed by heterologous expression in *P. pastoris* under GAP promoter for activity, pH stability and thermal stability (70°C) enhancements to obtain better XOS yield from xylan. Apart from these strategies, metagenomics approach is also very useful in obtaining novel enzymes with remarkable capabilities to produce oligosaccharides. For example, a thermophilic GH11 endo-β-1,4-xylanase from metagenomic library prepared from bagasse was used for XOS production from sugarcane biomass after its CBM trimming (X11C) and Pro71Thr mutation by random mutagenesis [163].

### 6.5 Biorefinery approach and integrated downstream processing

Biorefinery approach for prebiotic synthesis relies upon the cost-effective and sustainable valorization of various components of biomass into multiple valuable products, such as biofuels and biochemicals [84]. This approach has been successfully used for obtaining XOS, xylose and butanol from steam explosion pretreated *Eucalyptus* biomass, with 50% xylan and 80% glucan recovery via enzymatic hydrolysis at high solid loading [98]. Ion-exchange and resin treatment improved XOS recovery and enzymatic hydrolyzates used for butanol production by *Clostridium beijerinckii*. Martins et al. [149], used biorefinery approach for XOS (72.56% xylan conversion) and glucose production from sugarcane straw xylan and remaining glucan rich biomass, respectively. Coproduction of oligosaccharides XOS (xylobiose to xylohexose) and fermentable sugars by prehydrolysis of corncob by acetic acid hydrolysis (46%) followed by saccharification (91%) was carried out to achieve yields of ~140 g XOS, 328 g glucose, 25 g cellobiose, and 148 g xylose from 1 kg initial biomass [164]. Similarly, Álvarez et al. [165], obtained XOS (DP 2–6; 130 g/kg substrate), bioethanol (126 g/kg), and lignin (heating value of 23.4 MJ/kg) under biorefinery strategy employing custom-made enzyme cocktail of endoxylanase and accessory enzymes arabinofuranosidase, feruloyl-/acetylxylan-from steam explosion pretreated barley straw.

Removal of by-products and monomers via integrated approach of combined autohydrolysis, nanofiltration with discontinuous diafiltration and xylanase mediated enzymatic hydrolysis was adopted by Lian et al. [166], for high yield recovery of XOS (84%). Immobilization of recombinant endoxylanase of *B. subtilis* enzyme was attempted in a study by Milessi et al. [167], for increasing the enzyme recyclability up to 10 cycles achieving XOS (DP 2–4) production (20% xylan conversion) within 3 h without monomer accumulation. Ultrafiltration and nanofiltration based recovery of the COS from birch-biomass enhanced the product yield significantly [1].

### 6.6 Direct microbial fermentation of lignocellulose

An alternate to production and purification of microbial enzymes prior to enzymatic hydrolysis is direct microbial fermentation of LC biomass for potential prebiotic oligosaccharide synthesis. Using this approach, brewers’ spent grain was fermented by genetically modified *Bacillus subtilis* expressing a xylanase gene xyn2 from *T. reesei* to improve the AXOS production with yield of 54 mg/g and 33% increase in comparison to saccharification method. Using such approach, it is possible to save time, labor and cost associated with enzyme production. Similarly, Sharma et al. [155] used a GRAS bacterium *Leuconostoc mesenteroides* for biotransformation of sweet sorghum stalk extract into functional juice that is enrich with prebiotic oligosaccharides.

### 6.7 Use of statistical approaches

Application of statistical design of experiments in enhanced lignocellulose hydrolysis is not new [169]. Such approaches have been used recently to enhance the yield and titer of various oligosaccharides from LC biomass, e.g. orthogonal design for stepwise hydrolysis of bagasse for XOS and fermentable sugar production [151], Central Composite Rotatable Design (CCRD) for optimized XOS production by saccharification of sugarcane straw [149], CCRD for XOS (~73% xylan conversion) and glucose production from sugarcane straw by cocktail of in-house and recombinant enzyme [149], CCD and Box-Behnken design (BBD) for XOS synthesis from rice husk [170] and BBD-based response surface methodology for coproducing glucose and XOS from sugarcane bagasse [171].

### 6.8 Scale-up of LDOs production

Scarcity of the data on large-scale LDO production via controlled enzymatic hydrolysis of LCB and their scale-up, mainly due to commercial and economic interests of the producing industries, remained a big challenge in the past. Reports on scale-up of the production and purification processes of lignocellulose derived prebiotic oligomers have started to come only recently. In recent study, higher cellobiose production was reported at 100 mL level using birch and spruce biomass as feedstock, resulting in total C-2 production yield of 164 mg/g substrate, and 128 mg/g, respectively [75]. COS production process was recently successfully scaled-up from 0.020 L to nearly 100 g/L level, representing a 2.4× improvement [4]. Some other scale-up studies have been reported feasibility of COS production [172].

## 7. Challenges and future prospects of prebiotic oligomer production from lignocellulosic biomass

With the recent technological advancements described in this review, it is obvious that sustainable lignocellulose-derived prebiotics synthesis is gaining pace and it opens up several avenues that still remain untouched and need future explorations. There has been an increased global awareness about the health benefits of functional foods including prebiotic oligosaccharides, which have huge market potential. However, despite the advancements described in this review, there are many bottlenecks in the prebiotic oligosaccharide synthesis from lignocellulosic biomass using enzyme technology. Major challenges encountered during enzymatic LDO synthesis occur at feedstock/substrate level, during preprocessing or pretreatment, biochemical conversion, scale-up, pilot studies, etc.

At substrate level, the major challenges include collection, transportation, and storage of low-cost agro-industrial LCB feedstock to make it readily available to the producer industry throughout the year. Moreover, the biomass pretreatment is one of the major challenges, as there is no universal method applicable to all biomasses due to in their composition variations because of seasonal/climatic changes, different varieties of the agricultural crops, etc. So, efforts are needed in developing a single pretreatment method, ideally for a mixed feedstock containing two or more agro-residues obtained in different seasons, so that the biomass with similar composition can be used throughout the year, even in case on non-availability of a particular crop during a specific season.

Major hindrances in the sustainable and cost-effective conversion of LCB to oligosaccharides are related to biochemical conversion technology [173,174]. The foremost challenge is the selection of suitable enzyme source, as the lignocellulolytic enzymes are diverse and their activities must match the required application [175,176]. Therefore, bioprospecting of suitable environmental sources is highly recommended, so as to obtain suitable enzyme with desired capabilities, e.g. thermotolerance, pH tolerance, etc. Besides, unculturable and nonculturable microbial diversity need to be tapped from diverse environments for exploitation in prebiotic oligomer synthesis. This will require the use of ‘omics’ based approaches, including metagenomics, metatranscriptomics, and metaproteomics based studies for exploring better enzymes from the previously unexplored natural environments. Besides, the capabilities of the already existing enzymes need to be further enhanced by use of genomics and proteomics-based techniques, so as to improve their functions and make the enzymatic conversion of lignocellulosic biomass components more cost effective. Recombinant DNA technology has the capacity to enhance efficiency and stability of enzymes as per the requirements of the producing industry [177,178]. Robustness of the enzymes can be enhanced by various protein and enzyme engineering approaches, such as directed evolution and rational approaches. Moreover, due to lack of knowledge about structure-function relationships of oligosaccharide synthesis relevant enzymes, more research efforts need to be devoted in future on exploring the role and mechanisms of different structural domains/motifs of relevant enzymes. It will also open the gates for synthesis of customized cocktails for oligosaccharides generation, having more powerful prebiotic effects.

Synthetic biology has shown promise in developing ‘tailor-made’ or ‘designer’ microbes for biofuel, food and pharma applications, and its potential need to tapped in designing of customized cellular factories for enzyme production efficient bioconversion or even for direct fermentation of lignocellulosic substrate for prebiotic oligosaccharide biosynthesis. The bottleneck of economic recovery and purification of oligosaccharides need further interventions in development of low-cost, low effluent generating and faster downstream processing methods. Moreover, the enzyme immobilization and recovery through use of membrane-based technology can enhance the reusability of enzymes and significantly cut down costs of procurement or production of the enzymes.

Enzymes to be used in LDO synthesis, either obtained commercially or synthesized in-house, often contain other proteins and metabolic products synthesized by the enzyme producing microbe or medium components as impurities. Such materials should be present within limits and purities as per the prevailing good manufacturing practices. Recent developments in metabolic engineering and synthetic biology-based strategies for strain improvement have made it possible to enhance production of enzymes, which are safer-to-use and are functionally more efficient than their parental counterparts. Principally, wild-type and genetically modified microbes’ synthesized enzymes follow similar safety related guidelines, especially w.r.t. pathogenicity and toxigenic nature. However, currently recombinant enzymes are less commonly employed for prebiotic applications due to difficulties in their production scale-up and regulatory limitations. Recombinant enzymes need to be tested for transformable DNA encoding genes for antibiotic resistance. Moreover, necessary clearances from local and government agencies are needed before carrying out the genetic modifications of microbes. Biosafety as well acceptability of such enzyme preparations for prebiotic applications also the major concerns. Hopefully, future will witness the emergence of more recombinant enzymes in transforming biomass into prebiotic products [179].

Moreover, unlike GOS and FOS, LDOs are still not accepted as established prebiotics, as clinical trial studies on several LDOs are relatively less and recent research still does not provide exact reasons for their effects, which need future elucidations. For example, XOS are being used in some countries, but are still considered as ‘emerging prebiotics’ [61]. Among LDOs, XOS occupy the largest market share both in terms of quantity and price. As far as the regulatory considerations are concerned, only few XOS have received ‘GRAS’ status for food applications by US-Food and Drugs Administration. This is mainly due to the scarcity of data on human experiments for proving their worth in claimed benefits. Due to huge market potential of LDOs, especially XOS, currently more research and development efforts in terms of both investment and research intensification are much needed, especially for necessary approvals from government regulatory agencies and this will also help realize the actual commercial potential of LDOs [180]. Moreover, since prebiotics have a direct role in improving the growth and metabolism of gut organisms having potential probiotic properties, future research should focus on more applied aspects including symbiotic preparations and applications in improving human health. Gut microbiome-prebiotic interactions have been studied in details at molecular level, but such interactions in respect to all types of LDOs are still not known completely and need to be explored further, using the latest ‘omics’ tools.

Lastly, it is also crucial to assess the impact of enzyme technology for LDO synthesis on environment, society and economics. Therefore, life-cycle assessment of prebiotic oligosaccharide synthesis from lignocellulosic agro-industrial residues will be highly useful in assessing their environmental acceptability in a long run.

## 8. Conclusions

This review has provided a comprehensive and updated information on the enzymatic conversion of lignocellulosic biomass to prebiotic oligomers. Discussion on recent developments in enzymatic conversion technology for prebiotic oligomer synthesis indicated that this route is being increasingly employed for conversion of a wide variety of lignocellulosic resources, including agricultural wastes, forestry wastes, industrial wastes as well as marine wastes and is more sustainable than physico-chemical conversion route due to increased yield, lesser undesired products, mild operational conditions, and decreased cost. The recently used strategies for enhanced enzymatic conversion of lignocellulose to prebiotic oligomers such as tailor-made synergistic enzyme cocktails, enzyme reaction engineering, inhibition of specific enzyme activities, use of statistical designs and optimization techniques, direct microbial fermentation of lignocellulose, etc., were discussed in length. Heterologous expression and protein engineering approaches as well as biorefinery approach and integrated downstream processing have made it possible to enhance production and resource recovery from biomass. Lastly, an outlook for sustainable prebiotic oligomer production from biomass resources as well as the future research was provided, considering the challenges related to biomass, biochemical conversion technology, enzyme improvements, clinical acceptance, environmental and socio-economic benefits, etc. Based on the information provided in this review, it is evident that the enzyme technology has a huge potential in enhanced production of lignocellulose-derived oligosaccharides and with the advent of protein engineering, and other rational enzyme engineering approaches, as well as availability of custom-made enzyme cocktails, this field will grow exponentially in near future.

## Highlights

- Comprehensive review on enzymatic production of lignocellulosic-derived prebiotics
- Challenges of controlled enzymatic synthesis of lignocellulose
- Protein engineering for enhanced enzymatic prebiotic synthesis
- Recent approaches for sustainable production of biomass derived oligomers
- Market potential and scale-up aspects
